# The canacla, a new technology for hand washing

**DOI:** 10.1186/1753-6561-5-S6-O39

**Published:** 2011-06-29

**Authors:** B Vanhercke, J Vanhercke, A Vanhercke

**Affiliations:** 1 North-South Committee, Middelkerke, Belgium; 2 CHNU (Centre Hospitalier National Universitaire), Dakar, Senegal; 3 African Partnership for Patient Savety, Dakar, Senegal

## Introduction / objectives

By washing our hands under the tap, we use on average 3 litres of water (3,000 ml). This is an *enormous waste of water*, a precious natural resource which we should stop wasting.

## Methods

With the Canacla, we wash our hands better than under the tap, while using 30 times less water.

The Canacla revolutionizes an everyday gesture: washing hands is no longer a hidden act, done **a few** times a day, far away from other people: since the Canacla (an attractive and decorative object) is very accessible (because it’s placed in the busiest part of the house), "*I’m going* to wash my hands" is replaced by "*I’m washing my hands*, *right here*, *right now!*"Thus, hands are not only better washed, they are also more often washed.

## Results

1. the Canacla helps us to protect our health better

2. the Canacla contributes to sustainable development

2.1. With the Canacla, we can wash our hands properly while using 30 times less water, a precious natural resource which we should stop wasting.

2.2. Thirty times less water for hand washing also means 30 times less water wasted by hand washing. Water treatment is a polluting activity – a lot of CO2 is produced – using the Canacla will help reduce the carbon impact and carbon footprint of human activities.

3. The Canacla is economical

3.1. It decreases the overall “health bill” by reducing the contamination risk of diseases spread by insufficient hand washing.

3.2. It decreases the overall water bill.

## Conclusion

The Canacla is a new technology for hand washing, a technology which contributes to a better protection of our health and reinforces sustainable development.

## Disclosure of interest

None declared.

